# Neuroendocrine differentiation and serotonin expression in oesophageal adenocarcinomas after neoadjuvant therapy: correlation with clinicopathological features and outcome

**DOI:** 10.1111/his.15364

**Published:** 2024-11-11

**Authors:** David W. Dodington, Stefano Serra, Tim Bracey, Runjan Chetty, Klaudia M. Nowak

**Affiliations:** ^1^ Laboratory Medicine Program University Health Network Toronto Canada; ^2^ Department of Laboratory Medicine and Pathobiology University of Toronto Toronto Canada; ^3^ Royal Cornwall Hospitals NHS Trust Truro UK; ^4^ Deciphex Dublin Ireland

**Keywords:** neoadjuvant chemoradiation, neuroendocrine differentiation, oesophageal adenocarcinoma, serotonin

## Abstract

**Aims:**

Oesophageal adenocarcinoma (EAC) is a glandular or mucinous epithelial malignancy that can show immunohistochemical evidence of neuroendocrine differentiation (NED) and express the hormone serotonin. The objective of this study was to correlate the presence of NED and serotonin with clinicopathological characteristics and patient outcome after neoadjuvant chemoradiation.

**Methods and results:**

A retrospective cohort of patients treated between 2002 and 2021 was established and included 218 oesophagectomy specimens with residual tumour. Representative full‐face sections of tumour were stained for synaptophysin, chromogranin‐A and serotonin by immunohistochemistry, and staining results were correlated with disease‐free survival (DFS) and overall survival (OS). In total, 129 (59%) tumours showed evidence of NED, defined as immunohistochemical expression of synaptophysin or chromogranin‐A, while 40 (18%) showed evidence of NED and expressed serotonin. Patients with neuroendocrine‐positive tumours had significantly shorter median OS compared to those with neuroendocrine‐negative tumours (22.5 versus 48.8 months, *P* = 0.006), but similar median DFS (13.3 versus 17.8 months, *P* = 0.34). Using Cox regression, the association between NED and OS was significant in univariate [hazard ratio (HR) = 1.68, 95% confidence interval (CI) = 1.16–2.45] and multivariate (HR = 1.65, 95% CI = 1.08–2.52) analysis. Patients with serotonin‐expressing tumours had similar median OS (21.7 versus 25.9 months, *P* = 0.24) and DFS (7.3 versus 15.6 months, *P* = 0.12) compared to those with NED but lacking serotonin. Using Cox regression, serotonin expression was associated with reduced OS in univariate (HR = 1.62, 95% CI = 1.06–2.47) but not multivariate (HR = 1.03, 95% CI = 0.64–1.65) analysis.

**Conclusions:**

Our findings support NED as independent predictor of OS in EAC after neoadjuvant chemoradiation. While a subset of tumours with NED expressed serotonin, this did not provide additional prognostic information.

## Graphical abstract

Expression of neuroendocrine markers in oesophageal adenocarcinoma after neoadjuvant chemoradiation is independently associated with reduced overall survival. Assessment of neuroendocrine differentiation is a useful tool for predicting patient outcome.

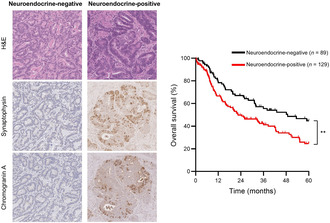

**Abbreviations**
DFS: Disease-free survival
EAC: Oesophageal adenocarcinoma
HR: Hazard ratio
INSM1: Insulinoma-associated protein 1
NED: Neuroendocrine differentiation
OS: Overall survival

## Introduction

Oesophageal adenocarcinoma (EAC) is a histological type of primary oesophageal cancer that is rapidly increasing in incidence in high‐income countries.^1^ Treatment of EAC with neoadjuvant chemoradiation provides survival benefit, particular in the setting of clinically positive lymph nodes.^2^ Despite this advance in therapy, most patients still have residual tumour and poor outcomes,^3^, ^4^ indicating a need to more clearly understand the pathology of EAC.

EAC, by definition, is an epithelial malignancy with glandular or mucinous differentiation.^5^ While retaining their distinctive exocrine phenotype, EAC can also show morphological or immunohistochemical evidence of neuroendocrine differentiation (NED). NED by immunohistochemistry typically presents as scattered non‐diffuse staining for neuroendocrine markers such as synaptophysin or chromogranin in a tumour that would otherwise be considered a conventional adenocarcinoma.^6^, ^7^ While few studies have investigated the clinical significance of NED in EAC, the presence of NED has been associated with shorter disease‐free survival (DFS) and overall survival (OS) after neoadjuvant chemoradiation.^7^

Serotonin (also known as 5‐hydroxytryptamine or 5‐HT) is a neurotransmitter with diverse functions and is predominantly secreted by enterochromaffin cells in the gut.^8^ Serotonin production has been described in well‐differentiated neuroendocrine tumours of the oesophagus^9^; however, expression of serotonin in EAC has yet to be reported. Our group has observed that a subset of EACs with NED also express serotonin, but the prevalence and clinical significance of this finding is currently unknown.

The overall objective of this study was to investigate the clinical significance of NED and serotonin expression in EACs after neoadjuvant chemoradiation. Specifically, the first aim was to confirm previous reports that NED is associated with shorter DFS and OS in a large independent patient cohort. The second aim was to determine the prevalence of serotonin expression in these tumours and to correlate this with clinicopathological characteristics and patient outcome.

## Materials and methods

### Patient cohort and case selection

A retrospective cohort of patients who received neoadjuvant chemoradiation for EAC (including tumours of the oesophagogastric junction) between the years 2002 and 2021 at the University Health Network (Toronto General Hospital, Princess Margaret Cancer Center) was established. Ethics approval was obtained from the University Health Network research ethics committee (21‐5284) and the study was performed in accordance with the Declaration of Helsinki. A total of 241 oesophagectomy specimens with residual tumour (treatment response score 1 or higher) were identified, 233 of which had original diagnostic material available for review. After review of the haematoxylin and eosin (H&E)‐stained slides, we excluded eight cases with insufficient tumour for immunohistochemistry (e.g. only rare single tumour cells present), and seven cases with residual intramucosal carcinoma (pT1a) only, leaving a total of 218 cases for inclusion in the study. One case showed distinct components of adenocarcinoma and large‐cell neuroendocrine carcinoma (confirmed by immunohistochemistry), and met criteria for classification as a mixed neuroendocrine–non‐neuroendocrine neoplasm. This case was not excluded from the study. Tumour regression scores were determined using the modified Ryan scheme (near complete response: single cells or rare small groups of cancer cells; partial response: residual cancer with evident tumour regression, but more than single cells or rare small groups of cancer cells; poor or no response: extensive residual cancer with no evident tumour regression).^10^, ^11^ Clinicopathological characteristics and survival outcomes were recorded from the patient's electronic medical record. DFS and OS were calculated from the time of surgery to time of clinical recurrence and death for DFS and OS, respectively. If unknown, the time of last follow‐up was used, after which the data were censored.

### Immunohistochemistry

One representative full‐face section of tumour from the post‐treatment oesophagectomy specimen was selected for staining. The slide with the largest area of tumour was chosen, except when there was higher grade tumour elsewhere, in which case the slide with the largest area of high‐grade tumour was chosen. Pretreatment biopsies were not stained for neuroendocrine markers, as previous studies showed no association between the presence of neuroendocrine cells in pretreatment biopsies and patient outcomes.^6^, ^7^ The formalin‐fixed paraffin‐embedded block was cut into 4‐μm‐thick sections and stained for synaptophysin (clone 27G12; Leica Biosystems, Concord, ON Canada; antigen retrieval: citrate, primary antibody incubation: 1:200 × 1 h); chromogranin A [polyclonal; Dako, Burlington, ON Canada; antigen retrieval: low‐temperature Tris ethylenediamine tetraacetic acid (EDTA) pH 9.0; primary antibody incubation: 1:1000 × 1 h] and serotonin (clone 5HT‐H209; Dako, antigen retrieval: pepsin, primary antibody incubation: 1:200 × 1 h). Stained slides were assessed using a light microscope and tumour cells with unequivocal cytoplasmic granular staining were deemed positive. Staining of tumour cells was recorded semiquantitatively throughout the entire section as follows: < 1, 1–10%, 11–50%, > 50%. NED was defined as any amount of positive staining for either synaptophysin, chromogranin A or both. Intramucosal staining was not included in the assessment given the common occurrence of mucosal endocrine cell micronests and single endocrine cells following neo‐adjuvant treatment, which could be mistaken for residual tumour.^12^

### Statistical analysis

Associations with clinicopathological variables were assessed using the χ^2^ test. Kaplan–Meier curves with the log‐rank test and Cox proportional hazards models were used to correlate NED and serotonin expression with DFS and OS. Statistical significance was defined as *P* < 0.05. Data analysis was performed using the SPSS version 26 statistical software package and survival curves were created using Prism GraphPad version 10.

## Results

### Immunohistochemistry results

**Figure 1:**
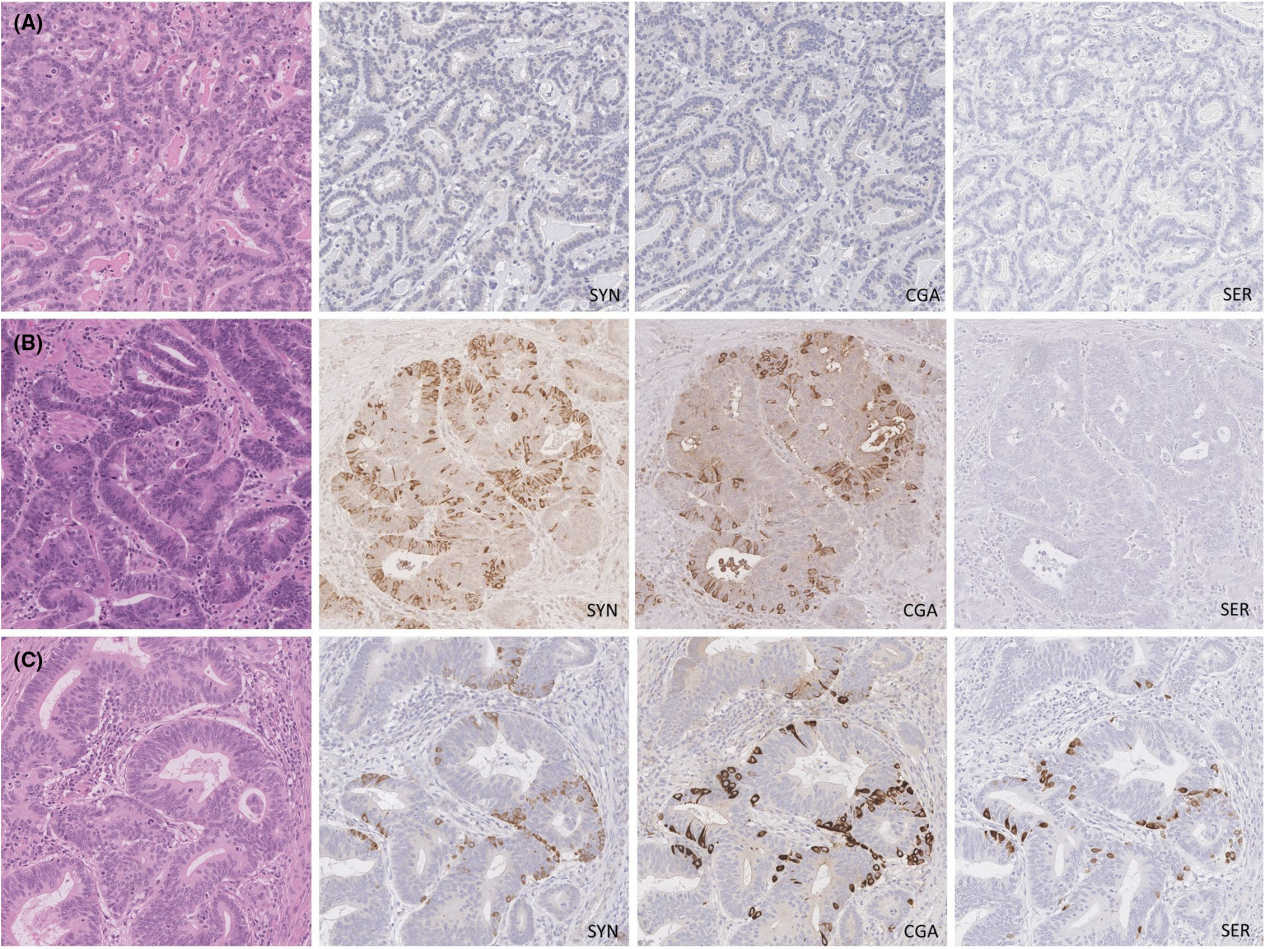
Representative immunohistochemical staining for neuroendocrine markers and serotonin in oesophageal adenocarcinomas after neoadjuvant chemoradiation. Haematoxylin and eosin (H&E) images (left column) and accompanying immunohistochemical stains for synaptophysin (SYN), chromogranin A (CGA) and serotonin (SER). **A**, Adenocarcinoma without neuroendocrine differentiation; **B**, adenocarcinoma with neuroendocrine differentiation but no serotonin expression. **C**, Adenocarcinoma with neuroendocrine differentiation and serotonin expression. [Color figure can be viewed at wileyonlinelibrary.com]

Of 218 cases, 129 (59%) showed evidence of NED. Among the cases with NED, 48 (37%) stained for synaptophysin only, five (4%) stained for chromogranin A only and 76 (59%) stained for both markers. Serotonin was expressed in 40 cases (31% of those with NED; 18% overall). Notably, serotonin was entirely absent in all cases lacking expression of either synaptophysin or chromogranin A. Representative staining results are shown in Figure 1 and the estimated extent of staining for each marker is shown in Table 1.

**Table 1:** Percentage of tumour cells staining positive for synaptophysin, chromogranin A and serotonin

	Synaptophysin	Chromogranin A	Serotonin
Negative	94 (43%)	137 (63%)	178 (82%)
Positive	124 (57%)	81 (37%)	40 (18%)
< 1%	29 (13%)	25 (12%)	21 (10%)
1–10%	65 (30%)	39 (18%)	13 (6%)
11–50%	14 (6%)	11 (5%)	6 (3%)
> 50%	16 (7%)	6 (3%)	0 (0%)

### Clinicopathological features

The clinicopathological characteristics by neuroendocrine status are shown in Table 2. There were no differences in patient age or sex. While chemotherapy protocols differed over the time‐span of the study, none of them were associated with higher prevalence of NED. Furthermore, the prevalence of NED was equal across time‐spans of the study. Pretreatment biopsies were available in 173 (79%) cases, which showed no difference in histological grade. Data for HER2 amplification and mismatch repair protein status were available in only a small number of pretreatment biopsies, but did not appear to correlate with NED. In the oesophagectomy specimens, there were no differences in histological grade, tumour stage or nodal stage between groups; however, there was a significantly lower proportion of neuroendocrine‐positive tumours with a near‐complete response to chemoradiation, and a higher proportion with a poor response to therapy. Clinicopathological characteristics among the serotonin‐expressing cases did not differ significantly from neuroendocrine‐positive cases lacking serotonin.

**Table 2:** Clinical and pathological characteristics

	Neuroendocrine‐negative (*n* = 89)	Neuroendocrine‐positive (*n* = 129)	*P‐*value
Sex			0.70
Male	77 (87%)	108 (84%)	
Female	12 (14%)	21 (16%)	
Age (years)			0.23
< 50	13 (15%)	21 (16%)	
50–70	66 (74%)	83 (64%)	
> 70	10 (11%)	25 (19%)	
Chemotherapy (years predominantly in use)			0.13
Cisplatin + Irinotecan (2003–2011)	16 (21%)	17 (15%)	
Cisplatin + 5‐FU (2005–2012)	17 (22%)	18 (16%)	
MAGIC (ECF/ECX) (2008–2018)	5 (7%)	14 (12%)	
CROSS (2013–2021)	33 (43%)	46 (40%)	
FLOT (2018–2021)	6 (8%)	14 (12%)	
Other	0 (0%)	6 (5%)	
Study period (years)			0.89
2002–06	9 (10%)	12 (9%)	
2007–11	21 (24%)	25 (19%)	
2012–16	23 (26%)	35 (27%)	
2017–21	36 (40%)	57 (44%)	
Histological grade (pre‐treatment biopsy)			0.87
Well to moderately differentiated	44 (63%)	63 (61%)	
Poorly differentiated	26 (37%)	40 (39%)	
HER2 status (pretreatment biopsy)			0.64
Amplified	8 (22%)	17 (27%)	
Not amplified	29 (78%)	47 (73%)	
Mismatch repair status (pre‐treatment biopsy)			1.00
MMR proteins intact	8 (89%)	24 (92%)	
MMR protein loss	1 (11%)	2 (8%)	
Histologic grade (resection)			1.00
Well to moderately differentiated	55 (62%)	79 (62%)	
Poorly differentiated	34 (38%)	49 (38%)	
ypT stage			0.07
ypT1	17 (19%)	19 (15%)	
ypT2	17 (19%)	18 (14%)	
ypT3	49 (55%)	90 (70%)	
ypT4	6 (7%)	2 (2%)	
ypN stage			0.48
ypN0	36 (40%)	39 (30%)	
ypN1	19 (21%)	32 (25%)	
ypN2	20 (23%)	32 (25%)	
ypN3	14 (16%)	26 (20%)	
Treatment effect			0.005
Near complete response	28 (32%)	20 (16%)*	
Partial response	47 (53%)	70 (54%)	
Poor or no response	14 (16%)	39 (30%)*	

Data represent counts (proportions). *Significant difference within a row (*P* < 0.05). CROSS, paclitaxel + carboplatin; ECF, epirubicin + cisplatin + 5‐fluorouracil; ECX, epirubicin + cisplatin + capecitabine; FLOT, fluorouracil + leucovorin + oxaliplatin + docetaxel.

### Clinical outcome

**Figure 2:**
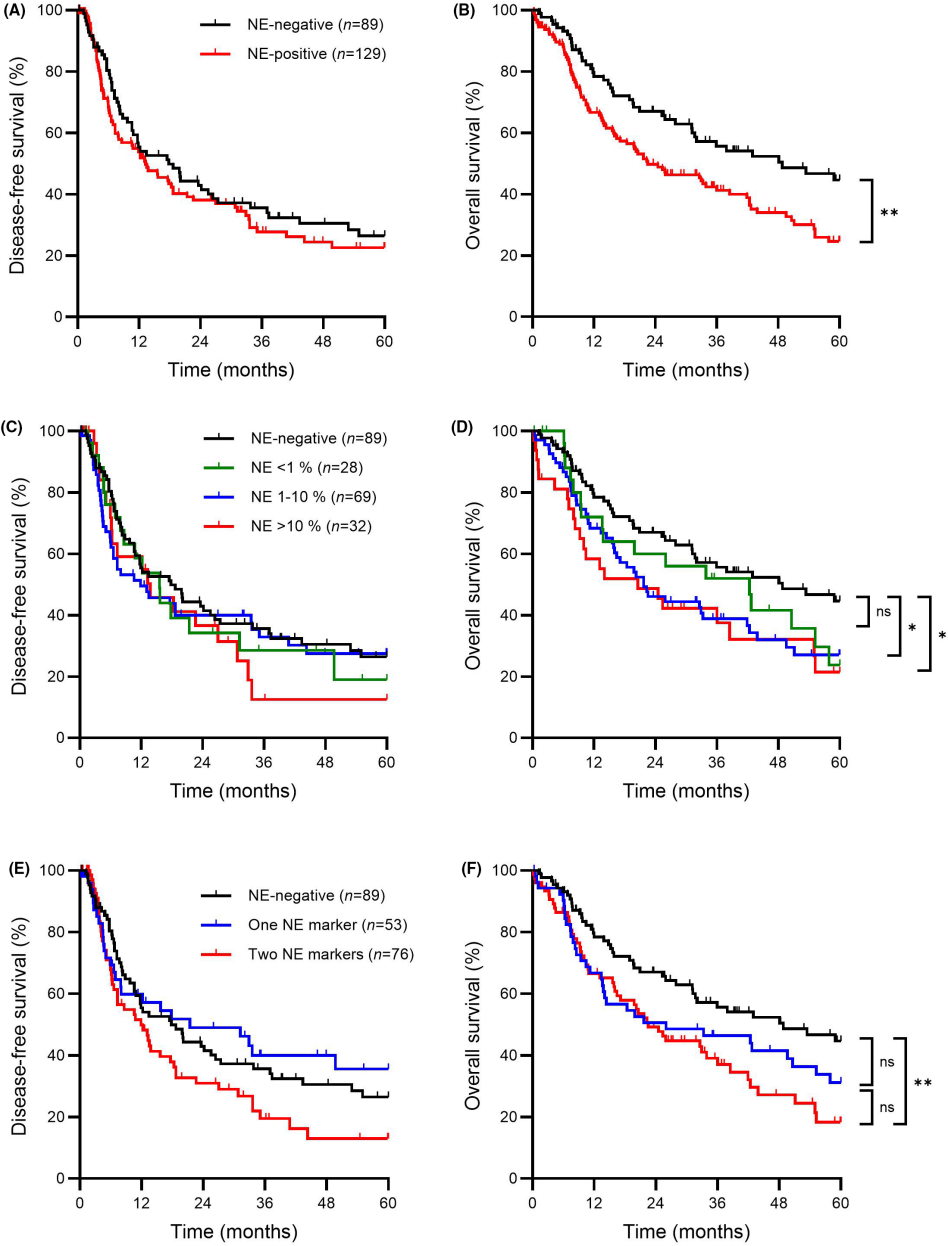
Kaplan–Meier curves of disease‐free survival and overall survival based on the presence of neuroendocrine differentiation. **A**, Disease‐free survival. **B**, Overall survival in patients with neuroendocrine‐negative tumours (*n* = 89, black line) and neuroendocrine‐positive tumours (*n* = 129, red line). **C**, Disease‐free survival; **D**, overall survival based on percentage of tumour cells staining positive for neuroendocrine markers; 0% (*n* = 89, black line), < 1% (*n* = 28, green line), 1–10% (*n* = 69, blue line) and > 10% (*n* = 32, red line). **E**, Disease‐free survival; **F**, overall survival in patients with neuroendocrine‐negative tumours (*n* = 89, black line), with tumours staining positive for one neuroendocrine marker (*n* = 53, blue line) and with tumours staining positive for two neuroendocrine markers (*n* = 76, red line). Statistical significance was determined using the log‐rank test. NE, neuroendocrine; ns, not significant; *P < 0.05, **P < 0.01. [Color figure can be viewed at wileyonlinelibrary.com]

Median DFS was similar between patients with neuroendocrine‐negative tumours and those with neuroendocrine‐positive tumours (17.8 versus 13.3 months, *P* = 0.34, Figure 2A); however, compared to patients with neuroendocrine‐negative tumours, those with neuroendocrine‐positive tumours had significantly shorter median OS (48.8 versus 22.5 months, *P* = 0.006, Figure 2B).

Subgroup analysis by extent of neuroendocrine marker staining was performed to evaluate for a possible dose–response relationship. Groups were formed using categories described in Table 1 based on the highest amount of either synaptophysin or chromogranin staining. Cases with 11–50% and > 50% neuroendocrine marker staining had similar outcomes and were combined into one group to more effectively balance the sample size for statistical analysis. Median DFS was similar between cases with 0%, < 1%, 1–10% and > 10% neuroendocrine marker staining (17.8 versus 15.6 versus 11.9 versus 13.8 months, respectively, overall *P* = 0.74, Figure 2C). There were, however, significant differences in median OS among cases with 0%, < 1%, 1–10% and >10 % neuroendocrine marker staining (48.8 versus 42.4 versus 21.7 versus 20.6 months, respectively, overall *P* = 0.033, Figure 2D). Compared to the neuroendocrine‐negative group, the difference in OS was statistically significant for both the 1–10% group (*P* = 0.19) and the > 10% group (*P* = 0.010). While OS for the < 1% group tended to be intermediate between the neuroendocrine‐negative and other groups, the differences were not statistically significant (neuroendocrine‐negative versus < 1%, *P* = 0.19; < 1% versus 1–10%, *P* = 0.31, < 1% versus >10%, *P* = 0.34).

Given that some definitions of NED require staining for two neuroendocrine markers, we also performed subgroup analysis according to the number of positive stains. Median DFS was similar between patients with neuroendocrine‐negative tumours; those with tumours staining positive for one neuroendocrine marker and those with tumours staining positive for two neuroendocrine markers (27.1 versus 30.1 versus 20.0 months respectively, overall *P* = 0.09, Figure 2E). Median OS differed significantly between patients with neuroendocrine‐negative tumours, those with tumours staining positive for one neuroendocrine marker and those with tumours staining positive for two neuroendocrine markers (39.2 versus 32.3 versus 28.9 months, respectively, overall *P* = 0.013, Figure 2F). Compared to the neuroendocrine‐negative group, the association was significant for the group with positive staining for two neuroendocrine markers (*P* = 0.003), but was just short of statistical significance for the group with positive staining for one neuroendocrine marker (*P* = 0.08). While those with staining for two markers tended to have shorter OS compared to those staining for one marker, the difference did not reach statistical significance (*P* = 0.32).

**Figure 3:**
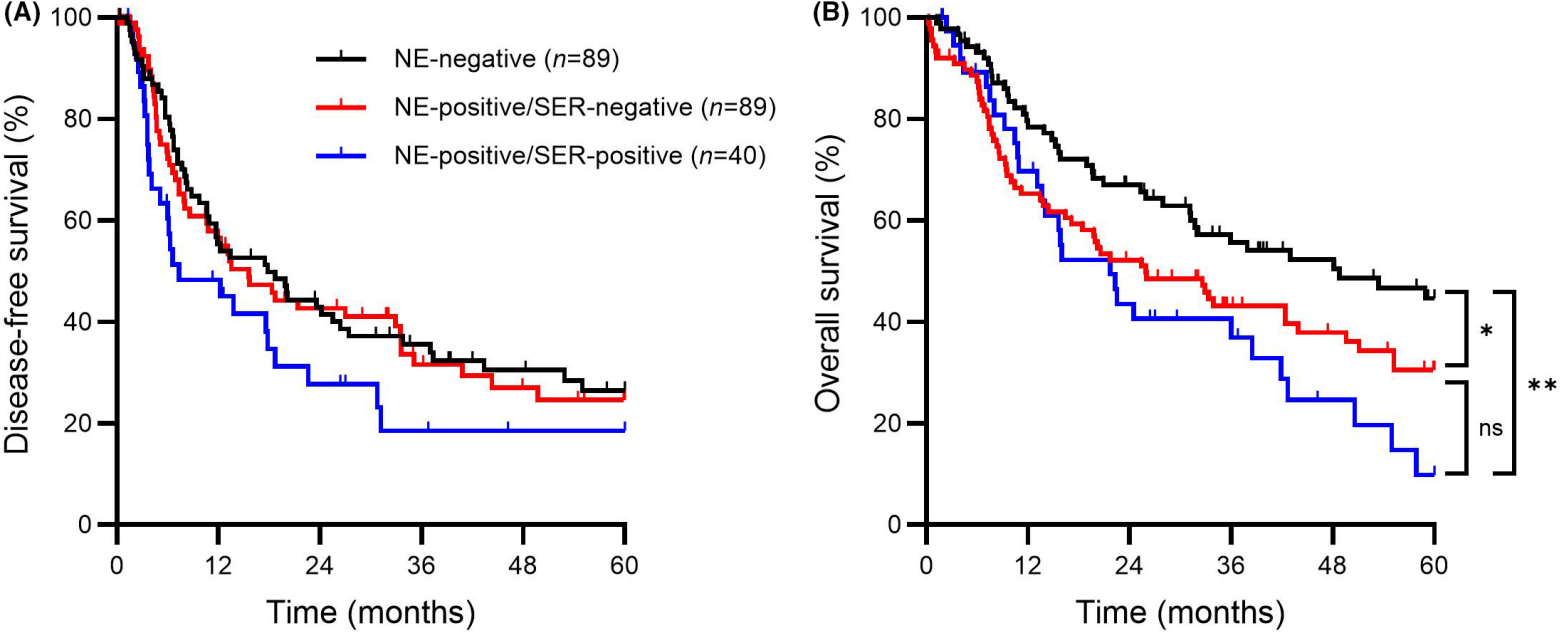
Kaplan–Meier curves of disease‐free survival and overall survival based on the presence of neuroendocrine differentiation and expression of serotonin. **A**, Disease‐free survival; **B**, overall survival in patients with neuroendocrine‐negative tumours (*n* = 89, black line), neuroendocrine‐positive/serotonin‐negative tumours (*n* = 89, red line) and neuroendocrine‐positive/serotonin‐positive tumours (*n* = 40, blue line). Statistical significance was determined using the log‐rank test. NE, neuroendocrine; ns, not significant; SER, serotonin; *P < 0.05, **P < 0.01. [Color figure can be viewed at wileyonlinelibrary.com]

Lastly, to account for serotonin expression, data were analysed as three groups. DFS was similar between neuroendocrine‐negative, neuroendocrine‐positive/serotonin‐negative and neuroendocrine‐positive/serotonin‐positive groups (17.8 versus 15.6 versus 7.3 months, respectively, overall *P* = 0.18, Figure 3A). Significant differences in OS, however, were seen among the same groups (48.8 versus 25.9 versus 21.7 months, respectively, overall *P* = 0.010, Figure 3B). Compared to the neuroendocrine‐negative group, the median OS was significantly shorter for both the neuroendocrine‐positive/serotonin‐negative and neuroendocrine‐positive/serotonin‐positive groups (*P* = 0.036 and *P* = 0.002, respectively). OS tended to be shorter in the neuroendocrine‐positive/serotonin‐positive group; however, the difference compared to the neuroendocrine‐positive/serotonin‐negative group was not statistically significant (*P* = 0.24).

Cox regression was then performed to assess the independent prognostic significance of NED and serotonin expression on patient survival. In univariate analysis, ypT stage, ypN stage and treatment response score were significant predictors of DFS (Table 3). Serotonin expression had a borderline, but not significant (*P* = 0.07) association with DFS. In the multivariate model, only ypN stage was independently prognostic of DFS. For OS, the presence of NED, serotonin expression, ypT stage and ypN stage were significant predictors in univariate analysis (Table 4). In the multivariate model, the presence of NED (but not serotonin), together with ypT stage and ypN stage, remained independently prognostic of OS.

**Table 3:** Cox proportional hazard model of disease‐free survival

	No.	Univariate			Multivariate		
		HR	95% CI	*P*‐value	HR	95% CI	*P*‐value
Neuroendocrine differentiation							
Absent (reference)	89						
Present	129	1.18	0.83–1.68	0.34	0.98	0.65–1.48	0.98
Serotonin expression							
Absent (reference)	178						
Present	40	1.49	0.96–2.29	0.07	1.28	0.78–2.09	0.33
Histological grade							
G1–G2 (reference)	134						
G3	83	1.11	0.78–1.59	0.60	1.03	0.71–1.50	0.87
ypT stage							
ypT1 (reference)	36						
ypT2	35	1.59	0.83–3.03	0.16	1.37	0.69–2.70	0.37
ypT3	139	1.87	1.11–3.16	0.018	1.42	0.78–2.56	0.25
ypT4	8	5.61	2.37–13.28	0.00009	2.62	0.98–7.01	0.055
ypN stage							
ypN0 (reference)	75						
ypN1	51	1.79	1.09–2.95	0.022	1.71	1.00–2.92	0.048
ypN2	52	2.25	1.38–3.66	0.001	2.16	1.27–3.66	0.004
ypN3	40	4.44	2.70–7.35	0.000000005	3.50	1.96–6.25	0.00002
Treatment effect							
Score 1 (reference)	48						
Score 2	117	1.71	1.05–2.78	0.032	1.05	0.59–1.86	0.87
Score 3	53	2.51	1.48–4.28	0.001	1.24	0.64–2.40	0.53

HR, hazard ratio; CI, confidence interval.

**Table 4:** Cox proportional hazard model of overall survival

	No.	Univariate			Multivariate		
		HR	95% CI	*P*‐value	HR	95% CI	*P*‐value
Neuroendocrine differentiation							
Absent (reference)	89						
Present	129	1.68	1.16–2.45	0.006	1.65	1.08–2.52	0.021
Serotonin expression							
Absent (reference)	178						
Present	40	1.62	1.06–2.47	0.026	1.03	0.64–1.65	0.91
Histological grade							
G1–G2 (reference)	134						
G3	83	1.28	0.89–1.84	0.18	1.22	0.83–1.79	0.31
ypT stage							
ypT1 (reference)	36						
ypT2	35	1.49	0.76–2.94	0.25	1.39	0.68–2.82	0.36
ypT3	139	1.79	1.03–3.11	0.038	1.54	0.83–2.86	0.17
ypT4	8	5.19	2.08–12.93	0.0004	4.50	1.64–12.36	0.004
ypN stage							
ypN0 (reference)	75						
ypN1	51	1.88	1.10–3.21	0.020	1.76	0.99–3.13	0.055
ypN2	52	3.08	1.87–5.08	0.00001	2.83	1.64–4.88	0.0002
ypN3	40	3.65	2.16–6.16	0.0000001	2.96	1.63–5.37	0.0004
Treatment effect							
Score 1 (reference)	48						
Score 2	117	1.49	0.93–2.40	0.10	0.87	0.50–1.53	0.63
Score 3	53	1.70	0.99–2.89	0.053	0.82	0.43–1.55	0.54

HR, hazard ratio; CI, confidence interval.

## Discussion

Our study is the largest to date to investigate the prognostic significance of NED in EACs after neoadjuvant chemoradiation and confirms that NED is an independent marker of aggressive tumour behaviour. The study is novel in that it is the first to examine the impact of serotonin expression by ECAs. While patients with serotonin‐expressing tumours tended to have shorter OS and DFS, the impact was not significantly worse compared to the presence neuroendocrine‐positive tumour cells alone. Furthermore, our study sought to determine a threshold for defining NED in EAC. While our main analysis included any amount neuroendocrine marker staining to define NED, the subgroup analysis showed that a significant association with OS is best seen with a threshold of neuroendocrine marker staining of at least 1% of tumour cells. Similarly, the relationship was strongest when tumours stained positive for more than one neuroendocrine marker.

Our findings are consistent overall with the those reported by Wang *et al*., who investigated the significance of NED in a cohort of 73 patients treated for EAC.^7^ While we did not see a relationship with DFS, both studies showed an independent association with OS, which is considered the ‘gold standard’ primary clinical end‐point.^13^ A different study by Hamilton *et al*. examined chromogranin A staining in 52 cases of EAC, but found no association with patient outcome.^6^ A potential explanation for the alternate conclusion could be the lower sensitivity of chromogranin A, as we found that 37% of tumours with NED stained for synaptophysin only, in keeping with the known superior sensitivity of synaptophysin.^14^ Additionally, biopsy samples were used, which could be less sensitive in detecting focal expression of neuroendocrine markers.

Although few studies have investigated the significance of NED in the oesophagus, there is a growing body of literature that recognises the importance of NED in adenocarcinomas of the gastrointestinal tract. NED in colorectal adenocarcinomas has been the most widely investigated, and several studies have linked immunohistochemical evidence of NED with outcomes such as higher pTNM stage and reduced OS.^15^, ^16^, ^17^, ^18^, ^19^, ^20^, ^21^ Similarly, a number of studies in gastric adenocarcinoma have associated NED with adverse clinicopathological variables and reduced OS.^22^, ^23^, ^24^, ^25^, ^26^

Biological mechanisms to explain the development and behaviour of ECAs with NED are currently unknown; however, potential mechanisms have begun to emerge from data at other sites in the gastrointestinal tract. In gastric adenocarcinomas, neuroendocrine cells often contain the same TP53 mutations and microsatellite changes as their neighbouring non‐neuroendocrine cells, suggesting a possible clonal origin of both cell‐types.^27^ This has led to speculation that a cancer cell with ‘stem cell‐like’ properties exists, which is able to give rise to both exocrine and endocrine phenotypes. Several mechanisms to explain the aggressive nature of tumours with NED have been proposed. Increased expression of vascular endothelial growth factor (VEGF) and increased microvessel density, leading to increased propensity for metastasis, has been reported in gastric^25^ and colorectal^19^ adenocarcinomas with NED. Recently, NED has been associated with increased PI3K‐AKT signalling in colorectal adenocarcinomas, which could theoretically be attributed to paracrine secretion of neuroendocrine granules.^28^ Lastly, it has been suggested that NED in gastric adenocarcinomas is associated with a more immunosuppressive tumour environment, as demonstrated by increased regulatory T cells and markers of T cell exhaustion.^29^

There are several outstanding questions remaining. It is unclear whether NED arises in EACs secondary to neoadjuvant chemoradiation. Wang *et al*., using paired pre‐ and post‐treatment samples, found an increase in the prevalence and extent of NED after treatment.^7^ This could be due to treatment‐related neuroendocrine transformation or chemoresistance of native neuroendocrine tumour cells. Endocrine cell pseudo hyperplasia in the non‐neoplastic atrophic glandular mucosa has also been reported in post‐treatment oesophagectomy specimens, probably owing to inherent resistance of neuroendocrine cells to chemoradiation effects.^12^ The association between NED and the treatment response scores seen in our study and in other studies^7^ would support a relationship between the presence of neuroendocrine tumour cells and chemoradiation resistance. Treatment‐induced neuroendocrine transformation is also likely to contribute to the number of adenocarcinomas with NED seen in our study. In a study of rectal carcinomas, for example, up to 67.9% of tumours treated with chemoradiation show cells with neuroendocrine phenotype compared to only 17.7% in untreated control resection specimens.^30^ The large increase in the number of tumours with any number of neuroendocrine cells is consistent with a treatment‐induced change compared to chemoresistance alone. This phenomenon is also well described in adenocarcinomas of the prostate^31^ and lung,^32^ suggesting that this is a common feature among adenocarcinomas. In our study, determination of the proportion of cases that might be treatment‐induced is limited, as neoadjuvant therapy is the standard of practice for locally advanced oesophageal cancer, and therefore there are few untreated oesophagectomy specimens available for comparison. As well as further elucidation of underlying mechanisms, there are other potential future directions based on the findings from this study. We did not evaluate specimens from recurrent or metastatic sites post‐oesophagectomy; therefore, it would be interesting to note whether recurrent disease shows significant NED, which would provide further support for the hypothesis that survival is impacted by the presence and extent of NED. Lastly, follow‐up studies are required to determine the predictive role of NED with respect to adjuvant therapy, as this could have important treatment implications for patients.

The current study has some limitations. The overall number of patients with serotonin‐expressing tumours was small, and therefore the study may have been underpowered to detect differences in survival outcomes for this subgroup. Similarly, cases with < 1% neuroendocrine marker staining showed a non‐significant trend towards worse OS. The lack of statistical significance could be related to sample size, and thus the significance of rare neuroendocrine cells is not entirely clear. While we used standard neuroendocrine markers to assess for NED, immunohistochemistry was limited to one section per case and the performance of newer neuroendocrine markers such insulinoma‐associated protein 1 (INSM1) was not tested. INSM1 is reported to be a highly sensitive and specific marker of NED, and has the benefit of being a nuclear stain. In one large study, however, there was no increase in sensitivity for detecting NED among neuroendocrine neoplasms with addition of INSM1 to a panel of markers that included both synaptophysin and chromogranin A, except in the case of neuroendocrine carcinomas, where the combination of INSM1/synaptophysin resulted in more positive cases than synaptophysin/chromogranin A (94.4 versus 91.6%).^14^ Among morphologically non‐neuroendocrine tumours, the number of cases showing positivity for at least two neuroendocrine markers was the same for synaptophysin/chromogranin A compared to synaptophysin/INSM1. Therefore, while INSM1 may have a slightly improved our sensitivity and specificity, its absence in our study is not likely to have altered the overall conclusions. Lastly, our study is limited due to the possibility of unaccounted‐for confounding variables, which cannot be entirely excluded due its retrospective nature.

In conclusion, our findings support NED as an independent predictor of OS after neoadjuvant chemoradiation. This study adds to the growing body literature that recognises the importance of NED to our understanding and management of EAC.

## Conflicts of interest

The authors have no relevant financial or other conflicts of interest to declare.

## Data Availability

The data that support the findings of this study are available from the corresponding author upon reasonable request.
